# Date Attachable Offline Electronic Cash Scheme

**DOI:** 10.1155/2014/216973

**Published:** 2014-05-18

**Authors:** Chun-I Fan, Wei-Zhe Sun, Hoi-Tung Hau

**Affiliations:** Department of Computer Science and Engineering, National Sun Yat-sen University, Kaohsiung 80424, Taiwan

## Abstract

Electronic cash (e-cash) is definitely one of the most popular research topics in the e-commerce field. It is very important that e-cash be able to hold the anonymity and accuracy in order to preserve the privacy and rights of customers. There are two types of e-cash in general, which are online e-cash and offline e-cash. Both systems have their own pros and cons and they can be used to construct various applications. In this paper, we pioneer to propose a provably secure and efficient offline e-cash scheme with date attachability based on the blind signature technique, where expiration date and deposit date can be embedded in an e-cash simultaneously. With the help of expiration date, the bank can manage the huge database much more easily against unlimited growth, and the deposit date cannot be forged so that users are able to calculate the amount of interests they can receive in the future correctly. Furthermore, we offer security analysis and formal proofs for all essential properties of offline e-cash, which are anonymity control, unforgeability, conditional-traceability, and no-swindling.

## 1. Introduction

Due to the rapid growth of the Internet and communication developments, electronic commerce has become much more popular and widely used than ever [1–8]. The mobile telecommunications have been developed from 2 G to 3.5 G. Furthermore, LTE Advanced, 4 G, and 5 G are being implemented to the market in recent years. With the convenience of mobile network, people can do shopping or electronic payments by using any devices with network capability instead of leaving home. As a result, electronic commerce has been emphasized nowadays. Electronic cash (e-cash) is definitely one of the most popular research topics among electronic commerce. E-cash and the traditional cash notes are very much alike except e-cash is digitized and used on Internet transactions; therefore, it is very important that e-cash be able to hold the accuracy, privacy, and all other security concerns.

A typical e-cash system usually consists of payers (customers), payees (shops), and a bank. There are two types of e-cash in general which are online e-cash [9–13] and offline e-cash [14–27]. Online e-cash system involves participation of the bank during transactions (the payment stage). Banks are able to check whether customers have double-spent the e-cash(s) or not, and if yes, banks can terminate the transactions at once. Thus, the bank has to be online during every transaction and it may lead to a bottleneck of the system. On the other hand, while banks do not participate in the payment stage of offline e-cash systems, double-spending check is only held during the deposit stage. Yet, the bank is set to be offline, but the system design is usually much more complicated than the online type and it may lead to a longer transaction time. Since both systems have their own pros and cons, they are used under different circumstances.

Extending online and offline e-cash systems, many e-cash schemes with other different features have been proposed over the years. For instance, e-cash can be stored compactly such that the space to store these e-cash is much reduced [15, 16], e-cash is generated by multiauthorities instead of one bank only [25], exact payments e-cash [13], recoverable e-cash which can be recovered when an e-cash is lost [26], and so on.

Based on the majority of the existing approaches, we summarize that a secure e-cash system should satisfy the following requirements.

- * Anonymity*: no one, except the judge, can obtain any information of the e-cash owner's identity from the contents of e-cash.
- * Unlinkability*: no one, except the judge, can link any e-cash payment contents.
- * Unforgeability*: no one, except the bank, can generate a legal e-cash.
- * Double-Spending Control*: banks should have the ability to check if the e-cash is double-spent or not. No e-cash is allowed to be spent twice or more in an e-cash system.
- * Conditional-Traceability*: the system should be able to trace and revoke the anonymity of users who violate any of the security rules so that they will receive penalties.
- * No-swindling*: no one, except the real owner, can spend a valid offline e-cash successfully.

In order to perform double-spending checks, banks have to store information of e-cash(s) in their database. Thus, the database of banks grows in direct proportion to the number of e-cash(s) withdrawn. Embedding an expiration date into each e-cash has been considered since it helps the banks to manage the database more easily. On the other hand, customers have to exchange their expired e-cash(s) with banks for new ones so as to keep the validity of the e-cash. Furthermore, customers will receive interest from banks after cash is deposited. In order to guarantee customers will receive the right amount of interest, it is necessary for customers to attach the deposit date to their e-cash(s) and the date cannot be modified by anyone else [11]. So far, there are a number of online e-cash schemes with an expiration date attachment [9, 11, 28]. However, there are very few offline approaches [21].

In this paper, we are going to propose an efficient date attachable offline e-cash scheme and provide formal proofs on essential properties to it in the random oracle model. Considering the practical needs, we pioneer to embed two kinds of date, which are expiration data and deposit date, to the offline e-cash. Moreover, we will offer an* E-cash renewal protocol* in our scheme (Section 3.2.5). Users can exchange their unused expired e-cash for a new one with another valid expiration date more efficiently. Compared with other similar works, our scheme is efficient from the aspect of considering computation cost.

The rest of this paper is organized as follows. In Section 2, we briefly review techniques employed throughout our scheme. Our proposed scheme is described in Section 3 in detail. Security proofs and analysis are covered in Section 4. Features and performance comparisons are made in Section 5, and the conclusion is given in Section 6.

## 2. Preliminaries

In this section, we briefly review techniques used in our date attachable offline e-cash scheme.

### 2.1. Chaum's Blind Signature Scheme

Blind signature was first introduced by Chaum [29]. It has been widely used in e-cash protocols since it has been proposed. A signer will not be able to view the content of the message while she/he is signing the message. Afterwards, a user can get a message with the signature of the signer by unblinding the signed message. The protocol is described as follows.

1. Initialization:
2. The signer randomly chooses two distinct large primes *p* and *q*, then computes *n* = *pq* and *ϕ*(*n*) = (*p* − 1)(*q* − 1). Afterwards, the signer selects two integers *e* and *d* at random such that *ed* ≡ 1(mod *ϕ*(*n*)). Finally, the signer publishes the public parameters (*e*, *n*) and a one-way hash function *H*.
3. User → Signer: *α*
4. The user chooses a message *m* and a random integer *r* in *Z*_n_*, then blinds the message by computing *α* = *r* ^*e*^ *H*(*m*)mod⁡ *n* and sends it to the signer.
5. Signer → User: *t*
6. After receiving *α*, the signer signs it with her/his private key *d* and sends it back to the user. The signed message will be *t* = *α* ^*d*^mod⁡ *n*.
7. Unblinding:
8. After receiving *t* from the signer, the user unblinds it by computing *s* = *r* ^−1^ *t*mod⁡ *n*. The signature-message pair is (*s*, *m*).
9. Verification:
10. The (*s*, *m*) can be verified by checking if *s* ^*e*^ ≡ *H*(*m*) (mod⁡ *n*) is true or not.

### 2.2. Chameleon Hashing Based on Discrete Logarithm

Chameleon hashing was proposed by Krawczyk and Rabin [30]. The chameleon hash function is associated with a one-time public-private key pair; it is a collision resistant function except for users who own a trapdoor for finding collision. Any user who knows the public key can compute the hashing, and for those who do not know the private key (trapdoor), it is impossible for them to find any two inputs which lead to the same hashing output. On the contrary, any user who knows the trapdoor can find the collision of given inputs. The construction of the chameleon hashing based on discrete logarithm is described as follows.

1. * Setup*:
  - *p*, *q*: two large primes such that *p* = *kq* + 1,
  - *g*: an element order *q* in *Z*_p_*,
  - *x*: private key in *Z*_q_*,
  - *y*: public key, where *y* = *g* ^*x*^ mod *p*.
2. * The function*: a message *m* ∈ *Z*_q_* is given and a random integer *r* ∈ *Z*_q_* is chosen. The hash is defined as cham-hash_*y*_(*m*, *r*) = *g* ^*m*^ *y* ^*r*^ mod *p*.
3. * Collision*: for a user who knows *x*, she/he is able to find the collision of the hash for any given *m*, *m*′ such that cham-hash_*y*_(*m*, *r*) =  cham-hash_*y*_(*m*′, *r*′). The user derives *r*′ in the equation *m* + *xr* = *m*′ + *xr*′ (mod *q*).

## 3. The Proposed Date Attachable Offline Electronic Cash Scheme

In this section, we will introduce a new date attachable offline e-cash scheme. Considering the issues mentioned in Section 1, we propose a secure offline e-cash scheme with two specific kinds of date attached to the e-cash, which are expiration date and deposit date.

### 3.1. Outline of the Proposed Scheme

Here we are going to briefly describe the procedures of our scheme. The proposed scheme contains four protocols,* withdrawal protocol, payment protocol, deposit protocol,* and* e-cash renewal protocol.* A user withdraws an e-cash with an expiration date attached to it from the bank. A trusted computing platform (i.e.,* judge device*) [31, 32], as stated in the proposed scheme, is installed in the bank to hold the identity information of all users and it will further help trace users when it is needed. It is impossible for anyone except the judge to obtain any information embedded in the device [33]. Nowadays, judge device can be implemented by the technique of Trusted Platform Module (TPM) [32, 34] in practice.

Before an e-cash is deposited, the depositor attaches the deposit date on the e-cash and sends it to the bank during the deposit stage. When the bank receives an e-cash, it will perform double-spending checking to verify whether the e-cash is doubly spent or not. The bank can derive secret parameters of the user who does double-spending and let the judge revoke the anonymity of the user. Besides, when an unused e-cash is expired, a user will be able to exchange it for a new one with a new expiration date. In our scheme, for the efficiency concerns, some of the unused parameters of users can remain unchanged while exchanging for a new valid e-cash. In the following sections, we will describe our scheme in detail.

### 3.2. The Proposed Scheme

Firstly, we define some notations as follows.

1. *H*_1_, *H*_2_, *H*_3_: three one-way hash functions, *H*_1_, *H*_2_, *H*_3_ : {0,1}* → {0,1}^*n*^.
2. *H*_4_, *H*_5_: two one-way hash functions, *H*_4_, *H*_5_ : {0,1}* → {0,1}^*q*^.
3. ${\tilde{E}}_{x}$, ${\tilde{D}}_{x}$: a secure symmetric cryptosystem. Plaintext is both encrypted and decrypted with a symmetric key *x*.
4. ${\hat{E}}_{pk},{\hat{D}}_{sk}$: a secure asymmetric cryptosystem. Plaintext is encrypted with a public key *pk* and decrypted with the corresponding private key *sk*.
5. (*pk*_*j*_, *sk*_*j*_): the public-private key pair of the judge.
6. (*e*_*b*_, *d*_*b*_): the public-private key pair of bank.
7. *Date*: expiration date. It represents an effective spending date of a withdrawn e-cash. Any e-cash withdrawn in the same period will have the same expiration date, and vice versa.
8. ID_*c*_: the identity of user *C*.
9. *l*_*k*_, *l*_*r*_: the security parameters.
10. *A judge device*: a tamper-resistant device which is issued by the judge. It is installed into the system of the bank. It is impossible to intercept or modify any information stored in the device.

#### 3.2.1. Initialization

Initially, the bank randomly chooses two distinct large primes (*p*_*b*_, *q*_*b*_) and computes RSA parameters *n*_*b*_ = *p*_*b*_ *q*_*b*_. It selects an integer *e*_*b*_ at random such that GCD(*ϕ*(*n*_*b*_), *e*_*b*_) = 1, where *ϕ*(*n*_*b*_) = (*p*_*b*_ − 1)(*q*_*b*_ − 1) and 1 < *e*_*b*_ < *ϕ*(*n*_*b*_). Then, it finds a *d*_*b*_ such that *e*_*b*_ *d*_*b*_ ≡ 1(mod *ϕ*(*n*_*b*_)). Secondly, it also chooses two other large primes *p* and *q* and two generators *g*_1_ and *g*_2_ of order *q* in *Z*_p_*. Then, the bank publishes $\left(n_{b},e_{b},p,q,g_{1},g_{2},pk_{j},H_{1},H_{2},H_{3},H_{4},H_{5},\tilde{E},\tilde{D},\hat{E},\hat{D}\right)$. Meanwhile, the judge embeds $\left(n_{b},e_{b},p,q,g_{1},g_{2},pk_{j},sk_{j},H_{1},H_{2},H_{3},H_{4},H_{5},\tilde{E},\tilde{D},\hat{E},\hat{D}\right)$ into a judge device and issues it to the bank.

#### 3.2.2. Withdrawal Protocol

**Figure 1:**
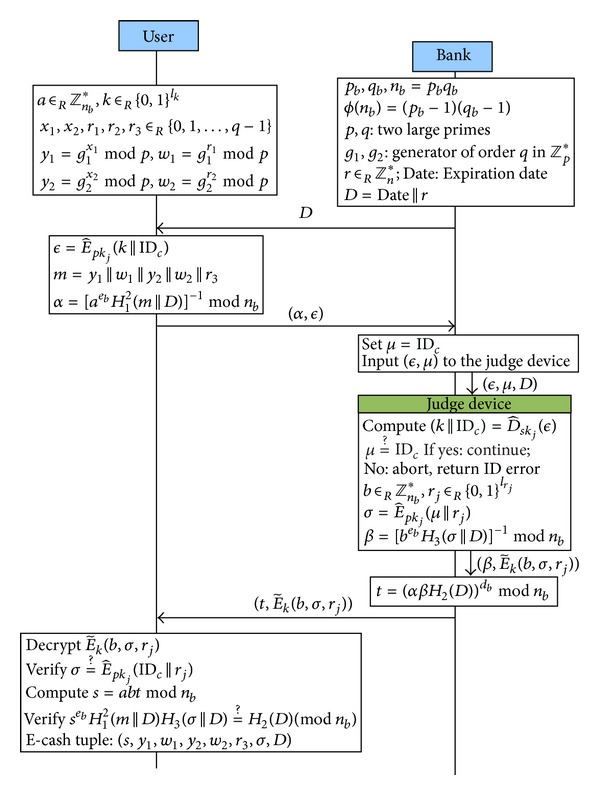
Withdrawal protocol.

Users run the withdrawal protocol with banks to get an e-cash, as shown in Figure 1, yet banks have to obtain information of users' identity, such as ID_*c*_ or account numbers, before the withdrawal protocol is proceeded. Therefore, users should perform an authentication with banks beforehand. Users can execute the withdrawal protocol by any devices that have the ability to compute and connect to the network. For instance, users can use mobile phones or computers to perform the withdrawal protocol and store the withdrawn e-cash. The detailed steps of the protocol are as follows.

(1) Bank → User: *D*
 Firstly, the user prepares parameters for withdrawing an e-cash. The user chooses integers *a*, *x*_1_, *x*_2_, *r*_1_, *r*_2_, and *r*_3_ in random, where *a* ∈_*R*_ *Z*_*n*_b__* and *x*_1_, *x*_2_, *r*_1_, *r*_2_, *r*_3_ ∈_*R*_ {0,1,…, *q* − 1} and selects a string *k* ∈_*R*_ {0,1}^*l*_*k*_^ randomly. The user then computes (*y*_1_, *w*_1_, *y*_2_, *w*_2_), where *y*_*i*_ = *g*_*i*_^*x*_*i*_^mod⁡ *p* and *w*_*i*_ = *g*_*i*_^*r*_*i*_^mod⁡ *p* for *i* = {1,2}. Secondly, the bank computes parameters for expiration date. It randomly chooses a *r* in *Z*_n_*, prepares *D* = Date||*r* for some expiration date *Date*. The bank will send *D* to the user when she/he requests to withdraw an e-cash.
(2) User → Bank: (*α*, *ϵ*)
 After receiving *D*, the user prepares $\epsilon ={\hat{E}}_{pk_{j}}(k||{\text{ID}}_{c})$ and
  (1) $$\begin{array}{c} \alpha ={\left[a^{e_{b}}H_{1}^{2}(m||D)\right]}^{-1}\mathrm{mod}n_{b}, \end{array}$$
 where *m* = (*y*_1_||*w*_1_||*y*_2_||*w*_2_||*r*_3_). Finally, the user sends (*α*, *ϵ*) to the bank.
(3) Bank → Judge device: (*ϵ*, *μ*, *D*)
 The bank sets *μ* = ID_*c*_, where ID_*c*_ is the identity of user *C*, and inputs it together with *ϵ* and *D* to the judge device.
(4) Judge device → Bank: $\left(\beta ,{\tilde{E}}_{k}(b,\sigma ,r_{j})\right)$
 The judge device decrypts *ϵ* and checks if *μ* = ID_*c*_. If not, it returns “ID error” to the bank; or else, it picks a random integer *b* ∈_*R*_ *Z*_*n*_b__* and a string *r*_*j*_ ∈_*R*_ {0,1}^*l*_*r*_*j*__^ randomly. Then it computes $\sigma ={\hat{E}}_{pk_{j}}(\mu ||r_{j})$ and
  (2) $$\begin{array}{c} \beta ={\left[b^{e_{b}}H_{3}(\sigma ||D)\right]}^{-1}\mathrm{mod}n_{b}. \end{array}$$
 Finally, it encrypts (*b*, *σ*, *r*_*j*_) by using the symmetric key *k* and outputs it together with *β* to the bank.
(5) Bank → User: $\left(t,{\tilde{E}}_{k}(b,\sigma ,r_{j})\right)$
 After receiving $\left(\beta ,{\tilde{E}}_{k}(b,\sigma ,r_{j})\right)$ from the judge device, it computes
  (3) $$\begin{array}{c} t={\left(\alpha \beta H_{2}\left(D\right)\right)}^{d_{b}}\mathrm{mod}n_{b} \end{array}$$
 and sends $\left(t,{\tilde{E}}_{k}(b,\sigma ,r_{j})\right)$ to the user.
(6) Verifications
 After receiving $\left(t,{\tilde{E}}_{k}(b,\sigma ,r_{j})\right)$, the user firstly decrypts the ciphertext by using the symmetric key *k* in order to obtain (*b*, *σ*, *r*_*j*_). Secondly, she/he checks if his/her ID is embedded correctly by computing if $\sigma ={\hat{E}}_{pk_{j}}(\text{I}{\text{D}}_{c}||r_{j})$ is true or not. Thirdly, she/he computes
  (4) $$\begin{array}{c} s=abt\mathrm{mod}n_{b} \end{array}$$

 

 and verifies *s* by checking if
  (5) $$\begin{array}{c} s^{e_{b}}H_{1}^{2}\left(m||D\right)H_{3}\left(\sigma ||D\right)=H_{2}\left(D\right)\left({\mathrm{mod}\;n}_{b}\right) \end{array}$$
 is true or not. Finally, when all verifications are done, the user gets the e-cash tuples (*s*, *m*, *σ*, *D*) and stores (*x*_1_, *x*_2_, *r*_1_, *r*_2_) for further payment usages.

#### 3.2.3. Payment Protocol

**Figure 2:**
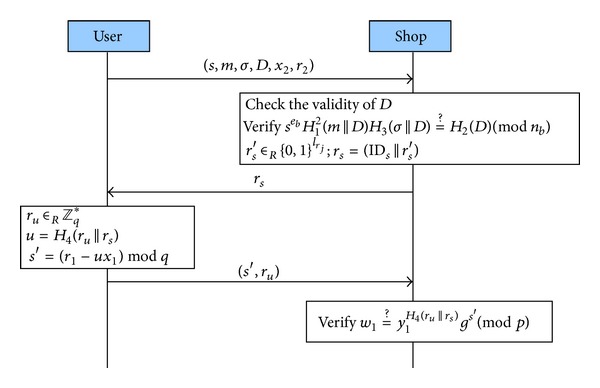
Payment protocol.

When a user has to spend the e-cash, she/he performs the protocol as shown in Figure 2. The steps of the protocol are described as follows.

(1) User → Shop: (*s*, *m*, *σ*, *D*, *x*_2_, *r*_2_)
 The user sends (*s*, *m*, *σ*, *D*, *x*_2_, *r*_2_) to the shop, where *D* contains the expiration date of the e-cash.
(2) Shop → User: *r*_*s*_
 The shop first checks *D* to verify if the e-cash is still within the expiration date or not. If not, it terminates the transaction. Otherwise, it continues to verify *s* ^*e*_*b*_^ *H*_1_^2^(*m*||*D*)*H*_3_(*σ*||*D*) = *H*_2_(*D*)(mod⁡ *n*_*b*_). If it is not valid, the protocol is aborted; or else, it selects a string *r*_*s*_′ ∈_*R*_ {0,1}^*l*_*r*_*j*__^ and sets a challenge *r*_*s*_ = (ID_*s*_||*r*_*s*_′), where ID_*s*_ is the identity of the shop. Finally, it sends *r*_*s*_ to the user.
(3) User → Shop: (*s*′, *r*_*u*_)
 After receiving *r*_*s*_ from the shop, the user randomly selects a *r*_*u*_ ∈_*R*_ *Z*_q_* and computes a response to the challenge
  (6) $$\begin{array}{c} s^{\prime }=\left(r_{1}-ux_{1}\right)\mathrm{mod}q, \end{array}$$
 where *u* = *H*_4_(*r*_*u*_||*r*_*s*_). Then, the user sends (*s*′, *r*_*u*_) to the shop.
(4) Verifications
 After receiving (*s*′, *r*_*u*_) from the user, the shop verifies if *w*_1_ = *y*_1_^*H*_4_(*r*_*u*_||*r*_*s*_)^ *g* ^*s*′^(mod⁡ *p*) is true or not. If it is true, the shop will accept the e-cash. On the other hand, if it is not, the shop will reject it. Since it is an offline e-cash, the shop does not have to deposit it to the bank immediately. It can store the e-cash and deposit it later together with other received e-cash(s).

#### 3.2.4. Deposit Protocol

**Figure 3:**
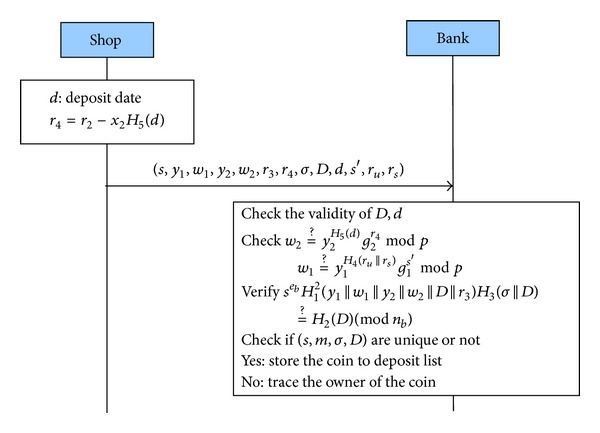
Deposit protocol.

As Figure 3 shows, shops attach the deposit date to their e-cash(s) and deposit them to banks in this protocol. Banks perform double-spending checks when they receive these e-cash(s). If any e-cash is double-spent, the bank will revoke the anonymity of the e-cash owner with the help of the judge. The steps are described in detail as follows.

(1) Shop → Bank: (*s*, *m*, *σ*, *D*, *d*, *r*_4_, *s*′, *r*_*u*_, *r*_*s*_)
 The shop computes *r*_4_ = *r*_2_ − *x*_2_ *H*_5_(*d*), where *d* is the deposit date, and sends (*s*, *m*, *σ*, *D*, *d*, *r*_4_, *s*′, *r*_*u*_, *r*_*s*_) to the bank.
(2) Verifications
 Firstly, the bank checks the correctness of expiration date *D* and deposit date *d*, respectively, and also checks if
  (7) w2=y2H5(d)g2r4mod⁡p,w1=y1H4(ru||rs)g2s′mod⁡p
 are true or not. Secondly, the bank verifies if *s* ^*e*_*b*_^ *H*_1_^2^(*m*||*D*)*H*_3_(*σ*||*D*) = *H*_2_(*D*)(mod⁡ *n*_*b*_) and checks the uniqueness of (*s*, *m*, *σ*, *D*). Finally, if all of the above facts are verified successfully, the bank will accept and store the e-cash in its database and record *H*_1_(*m*||*D*) in* exchange list*. Otherwise, it will reject this transaction and trace the owner of the e-cash.

#### 3.2.5. E-Cash Renewal Protocol

**Figure 4:**
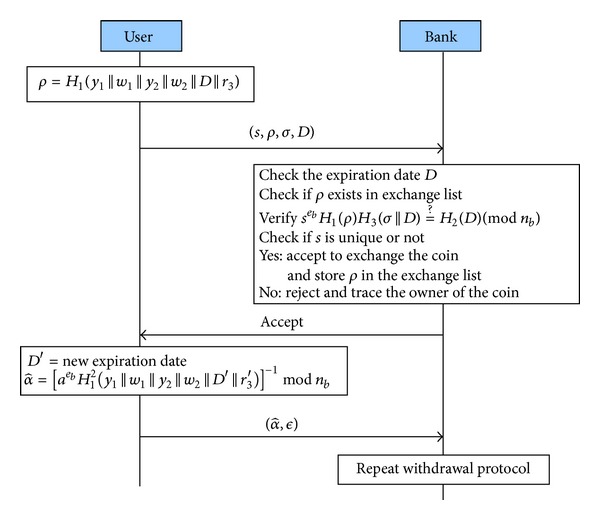
E-Cash renewal protocol.

In order to reduce the unlimited growth database problem of the bank, we have expiration date and renewal protocol in our scheme to achieve it, as shown in Figure 4. When an unused e-cash is expired, the user has to exchange it for another e-cash with a new expiration date from the bank.

(1) User → Bank: (*s*, *ρ*, *σ*, *D*)
 The user recalls *m* = (*y*_1_, *w*_1_, *y*_2_, *w*_2_, *x*_2_, *r*_3_) and prepares
  (8) $$\begin{array}{c} \rho =H_{1}\left(m||D\right) \end{array}$$
 and sends it together with the unused (*s*, *σ*, *D*) to the bank.
(2) Verifications
 Firstly, the bank checks the correctness of expiration date *D* and makes sure *ρ* does not exist in the* exchange list*. Secondly, the bank verifies if *s* ^*e*_*b*_^ *H*_1_(*ρ*)*H*_3_(*σ*||*D*) ≡ *H*_2_(*D*)(mod⁡ *n*_*b*_). Finally, if all of the above facts are verified successfully, the bank will accept to exchange the e-cash. It will send a new expiration date *D*′ and store *ρ* in the* exchange list*. Otherwise, it will reject the exchange request.
(3) User → Bank: $\left(\hat{\alpha },\epsilon \right)$
 The user computes
  (9) $$\begin{array}{c} \hat{\alpha }={\left[a^{e_{b}}H_{1}^{2}(m^{\prime }||D^{\prime })\right]}^{-1}\mathrm{mod}n_{b}, \end{array}$$
 where *m*′ = (*y*_1_, *w*_1_, *y*_2_, *w*_2_, *x*_2_, *r*_3_′), *r*_3_′ is a random, and *D*′ is the new expiration date issued by the bank. The user sends $\left(\hat{\alpha },\epsilon ,\text{I}{\text{D}}_{c}\right)$ to the bank. Then the bank repeats the withdrawal protocol in Section 3.2.2 from Step 2 with the user.

#### 3.2.6. Double-Spending Checking and Anonymity Control

In our scheme, the identity of the users is anonymous in general except when the users violate any security rules and, therefore, their identities will be revealed.

(1) Double-Spending Checking
 When an e-cash is being doubly spent, there must be two e-cash(s) with the same record prefixed by (*s*, *y*_1_, *w*_1_, *y*_2_, *w*_2_, *r*_3_, *σ*, *D*) stored in the database of the bank. Therefore, the bank is able to detect any double-spent e-cash easily by checking the above parameters. For instance, the bank has received two e-cash(s),
  (10) $$\begin{array}{c} \left(s,y_{1},w_{1},y_{2},w_{2},x_{2},r_{3},r_{4},\sigma ,D,d,s^{\prime },r_{u},r_{s}\right),\\ \left(s,y_{1},w_{1},y_{2},w_{2},x_{2},r_{3},\hat{r_{4}},\sigma ,D,\hat{d},\hat{s^{\prime }},{\hat{r}}_{u},{\hat{r}}_{s}\right). \end{array}$$
 Thus, the bank can obtain two equations as follows:
  (11) s′≡r1−H4(ru||rs)x1(mod⁡ q),s′^≡r1−H4(r^u||r^s)x1(mod⁡ q).
 The bank can derive (*x*_1_, *r*_1_) from the above equations and send (*s*, *y*_1_, *w*_1_, *y*_2_, *w*_2_, *x*_2_, *r*_3_, *σ*, *D*) and (*x*_1_, *r*_1_) to the judge to trace the owner of the e-cash.
(2) Revocation
 The judge can trace any user who doubly spends e-cash(s) or violates any transaction regulations. When the judge receives (*s*, *y*_1_, *w*_1_, *y*_2_, *w*_2_, *x*_2_, *r*_3_, *σ*, *D*) and (*x*_1_, *r*_1_) from the bank, it checks the following equations:
  (12) $$\begin{array}{c} s^{e_{b}}H_{1}^{2}\left(m||D\right)H_{3}\left(\sigma ||D\right)\overset{?}{\equiv }H_{2}\left(D\right)\left(\mathrm{mod}\;n_{b}\right),\\ y_{1}\;\overset{?}{\equiv }g_{1}^{x_{1}}\left(\mathrm{mod}\;p\right),\\ w_{1}\;\overset{?}{\equiv }g_{1}^{r_{1}}\left(\mathrm{mod}\;p\right). \end{array}$$
 If all of the above equalities are true, the judge will decrypt *σ* and return the extracted ID_*c*_ to the bank.

## 4. Security Proofs

In this section, we provide security definitions and formal proofs of the following security features: unlinkability, unforgeability, traceability, and no-swindling for our proposed date attachable offline electronic cash scheme (*DAOECS*).

### 4.1. E-Cash Unlinkability

Based on the definition of unlinkability introduced by Abe and Okamoto [35] and Juels et al. [36], we formally define the unlinkability property of *DAOECS*.

**Definition 1 (The Linkage Game)**

**Figure 5:**
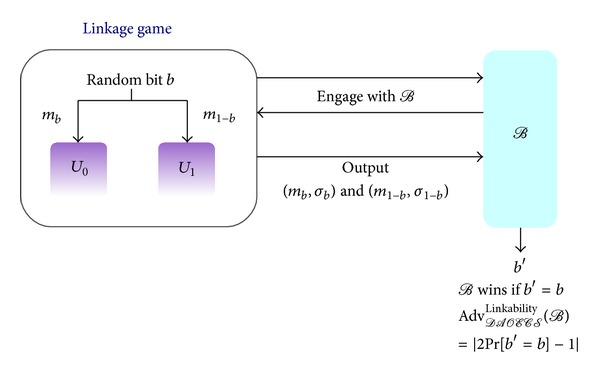
The game environment of linkage game.

Let *U*_0_, *U*_1_, and *J* be two honest users and the judge that follows *DAOECS*, respectively. Let *B* be the bank that participates the following game with *U*_0_, *U*_1_, and *J*. The game environment is shown in Figure 5.

**Step 1**

According to *DAOECS*, *B* generates the bank's public key (*e*_*b*_, *n*_*b*_), the bank's private key (*d*_*b*_, *p*_*b*_, *q*_*b*_), system parameters (*p*, *q*, *g*_1_, *g*_2_), the expiration date *D*, and the five public one-way hash functions *H*_1_, *H*_2_,  *H*_3_, *H*_4_, and *H*_5_. *J* generates the judge's public-private key pair (*pk*_*j*_, *sk*_*j*_).

**Step 2**

*B* generates *x*_1__*i*_, *x*_2__*i*_, *r*_1__*i*_, *r*_2__*i*_, *r*_3__*i*_ in random, where *x*_1_, *x*_2_, *r*_1_, *r*_2_, *r*_3_ ∈_*R*_ {0,1,…, *q* − 1}, and computes (*y*_*k*__*i*_, *w*_*k*__*i*_) for *k* = {1,2} and *i* = {0,1}, where *y*_*k*__*i*_ = *g*_*k*_^*x*_*k*_^mod⁡ *p* and *w*_*k*__*i*_ = *g*_*k*_^*r*_*k*_^mod⁡ *p*.

**Step 3**

We choose a bit $\hat{b}\;\in \;\{0,1\}$ randomly and place $\left({y_{1}}_{\hat{b}},{w_{1}}_{\hat{b}},{y_{2}}_{\hat{b}},{w_{2}}_{\hat{b}}\right)$ and $\left({y_{1}}_{1-\hat{b}},{w_{1}}_{1-\hat{b}},{y_{2}}_{1-\hat{b}},{w_{2}}_{1-\hat{b}}\right)$ on the private input tapes of *U*_0_ and *U*_1_, respectively, where $\hat{b}$ is not disclosed to *B*.

**Step 4**

*B* performs the withdrawal protocol of *DAOECS* with *U*_0_ and *U*_1_, respectively.

**Step 5**

If *U*_0_ and *U*_1_ output two e-cash(s) $\left(s_{\hat{b}},m_{\hat{b}},\sigma_{\hat{b}},D_{\hat{b}}\right)$ and $\left(s_{1-\hat{b}},m_{1-\hat{b}},\sigma_{1-\hat{b}},D_{1-\hat{b}}\right)$, where *m*_*i*_ = (*y*_1_*i*__, *w*_1_*i*__, *y*_2_*i*__, *w*_2_*i*__, *r*_3_*i*__), on their private tapes, respectively, we give the two e-cash(s) in a random order to *B*; otherwise, ⊥ is given to *B*.

**Step 6**

*B* outputs ${\hat{b}}^{\prime }\in \{0,1\}$ as the guess of $\hat{b}$. The bank *B* wins the game if ${\hat{b}}^{\prime }=\hat{b}$ and *J* has not revoked the anonymity of $\left(s_{\hat{b}},m_{\hat{b}},\sigma_{\hat{b}},D_{\hat{b}}\right)$ and $\left(s_{1-\hat{b}},m_{1-\hat{b}},\sigma_{1-\hat{b}},D_{1-\hat{b}}\right)$ to *B*. We define the advantage of *B* as

(13) $$\begin{array}{c} \text{Ad}{\text{v}}_{DAOECS}^{\text{Linkability}}\left(B\right)=\left|2\text{Pr}\left[{\hat{b}}^{\prime }=\hat{b}\right]-1\right|, \end{array}$$

where $\text{Pr}[{\hat{b}}^{\prime }=\hat{b}]$ denotes the probability of ${\hat{b}}^{\prime }=\hat{b}$.

**Definition (Unlinkability)**

A *DAOECS* satisfies the unlinkability property if and only if the advantage Adv_*DAOECS*_^Linkability^(*B*) defined in Definition 1 is negligible.

**Theorem 3**

A *DAOECS* satisfies the unlinkability property of Definition 2 if the adopted cryptosystems are semantically secure.

**Proof**

If *B* is given ⊥ in the Step 5 of the game, it will determine $\hat{b}$ with probability 1/2, which is exactly the same as a random guess of $\hat{b}$.

Here, we assume that *B* gets two e-cash (*s*_0_, *m*_0_, *σ*_0_, *D*_0_) and (*s*_1_, *m*_1_, *σ*_1_, *D*_1_). Let $\left(\alpha_{i},\beta_{i},t_{i},\epsilon_{i},{\tilde{E}}_{k_{i}}(b_{i},\sigma_{i},r_{j_{i}})\right)$, *i* ∈ {0,1}, be the view of data exchanged between *U*_*i*_ and *B* in the withdrawal protocol (Section 3.2.2) and let (*x*_2_*i*__, *r*_2_*i*__, *r*_4_*i*__, *r*_*u*_*i*__, *r*_*s*_*i*__, *s*_*i*_′, *d*_*i*_) be the view of data exchanged when *B* performs the payment protocol (Section 3.2.3) and the deposit protocol (Section 3.2.4) by using (*s*_*i*_, *m*_*i*_, *σ*_*i*_, *D*_*i*_), where *i* ∈ {0,1}.

For (*s*, *m*, *σ*, *D*, *x*_2_, *r*_2_, *r*_4_, *r*_*u*_, *r*_*s*_, *s*′, *d*)∈

(14) {(s0,m0,σ0,D0,x20,r20,r40,ru0,rs0,s0′,d0),(s1,m1,σ1,D1,x21,r21,r41,ru1,rs1,s1′,d1)}

and $\left(\alpha_{i},\beta_{i},t_{i},\epsilon_{i},{\tilde{E}}_{k_{i}}(b_{i},\sigma_{i},r_{j_{i}})\right)$, *i* ∈ {0,1}, there always exists a pair (*a*_*i*_′, *b*_*i*_′) such that

(15) ai′=[αiH12(m||D)]−dbmod⁡nb (via(1)),bi′=[βiH3(σ||D)]−dbmod⁡nb (via(2)).

And from (3), *t*_*i*_ ≡ (*α*_*i*_ *β*_*i*_ *H*_2_(*D*))^*d*_*b*_^(mod⁡ *n*_*b*_), (4) always holds as

(16) s≡(ai′bi′ti)≡[(H12(m||D)H3(σ||D))−1H2(D)]db(mod⁡  nb).

Besides, ${\hat{E}}_{pk_{j}}$ and ${\tilde{E}}_{k_{i}}$ are semantically secure encryption functions. *B* cannot learn any information from *ϵ*_*i*_ and ${\tilde{E}}_{k_{i}}(b_{i},\sigma_{i},r_{j_{i}})$.

From the above, given any (*s*, *m*, *σ*, *D*)∈{(*s*_0_, *m*_0_, *σ*_0_, *D*_0_), (*s*_1_, *m*_1_, *σ*_1_, *D*_1_)} and (*α*_*i*_, *β*_*i*_, *t*_*i*_), where *i* ∈ {0,1}, there always exists a corresponding pair (*a*_*i*_′, *b*_*i*_′) such that (1), (2), (3), and (4) are satisfied.

Thus, go back to Step 6 of the game, the bank *B* succeeds in determining $\hat{b}$ with probability (1/2) + *ε*, where *ε* is negligible since $\hat{E}$ and $\tilde{E}$ are semantically secure. Therefore, we have Adv_*DAOECS*_^Linkability^(*B*) = 2*ε*, which is negligible, so that *DAOECS* satisfies the unlinkability property.

### 4.2. E-Cash Unforgeability

In this section, we will formally prove that the proposed date attachable offline electronic cash scheme (*DAOECS*) is secure against forgery attack. The forgery attack can be roughly divided into two types, one is the typical one-more forgery type (i.e., (*l*, *l* + 1)-forgery) [37] and the other is the forgery on some specific expiration date of an e-cash after sufficient communications with the signing oracle (i.e., bank). The details of definitions and our formal proofs will be described as follows.

**Definition 4 (Forgery Game 1 in *DAOECS* (FG-1))**

Let *l*_*k*_ ∈ *N* be a security parameter and *A* be an adversary in *DAOECS*. *O*_*S*_ is an oracle which plays the role of the bank in *DAOECS* to be responsible for issuing e-cash(s) (i.e., (*s*, *m*, *σ*, *D*), where *m* = (*w*_1_, *y*_1_, *w*_2_, *y*_2_, *r*_3_, *D*)) according to the queries from *A*. *A* is allowed to query *O*_*S*_ for *l* times; consider the experiment Exp_*A*_^FG-1^(*l*_*k*_) shown in Algorithm 1.  *A* wins the forgery game FG-1 if the probability Pr[Exp_*A*_^FG-1^(*l*_*k*_) = 1] of *A* is nonnegligible.

**Definition 5 (Forgery Game 2 in *DAOECS* (FG-2))**

Let *l*_*k*_ ∈ *N* be a security parameter and *A* be an adversary in *DAOECS*. *O*_*S*_ is an oracle which plays the role of the bank in *DAOECS* to take charge of the following two events:

- issue e-cash(s) (i.e., (*s*, *m*, *σ*, *D*), where *m* = (*w*_1_, *y*_1_, *w*_2_, *y*_2_, *r*_3_, *D*)) according to the queries from *A*,
- record the total number *l*_*D*_*i*__ of each distinct expiration date *D*_*i*_.

*A* is allowed to query *O*_*S*_ for *l* times; consider the experiment Exp_*A*_^FG-2^(*l*_*k*_) shown in Algorithm 2. *A* wins the forgery game FG-2 if the probability Pr[Exp_*A*_^FG-2^(*k*) = 1] of *A* is nonnegligible.

Here we introduce the hard problems used in our proof models.

**Definition 6 (Alternative Formulation of RSA Chosen-Target Inversion Problem (RSA-ACTI))**

Let *k* ∈ *N* be a security parameter and *A* be an adversary who is allowed to access the RSA-inversion oracle *O*_inv_ and the target oracle *O*_*t*_. *A* is allowed to query *O*_*t*_ and *O*_inv_ for *m* and *q*_*h*_ times, respectively. Consider Algorithm 3.

We say *A* breaks the RSA-ACTI problem if the probability Pr[Exp_*A*_^RSA-ACTI^(*k*) = 1] of *A* is nonnegligible.

**Definition 7 (The RSA Inversion Problem)**

Given (*e*, *n*), where *n* is the product of two distinct large primes *p* and *q* with roughly the same length and *e* is a positive integer relatively-prime to (*p* − 1)(*q* − 1), and a randomly-chosen positive integer *y* less than *n*, find an integer *x* such that *x* ^*e*^ ≡ *y* (mod⁡  *n*).

**Definition 8 (E-Cash Unforgeability)**

If there exists no probabilistic polynomial-time adversary who can win FG-1 or FG-2, then *DAOECS* is secure against forgery attacks.

**Theorem 9**

For a polynomial-time adversary *A* who can win FG-1 or FG-2 with nonnegligible probability, there exists another adversary *S* who can break the RSA-ACTI problem or RSA inversion problem with nonnegligible probability.

**Proof**

*S* simulates the environment and controls three hash oracles, *O*_*H*_1__, *O*_*H*_2__, *O*_*H*_3__ and an e-cash producing oracle *O*_*S*_ of *DAOECS* scheme to respond to different queries from *A* in the random oracle model and takes advantage of *A* to solve RSA-ACTI problem or RSA inversion problem, simultaneously. Then, for consistency, *S* maintains three lists *L*_*H*_1__, *L*_*H*_2__, and *L*_*H*_3__ to record every response of *O*_*H*_1__, *O*_*H*_2__, and *O*_*H*_3__, respectively.

**Figure 6:**
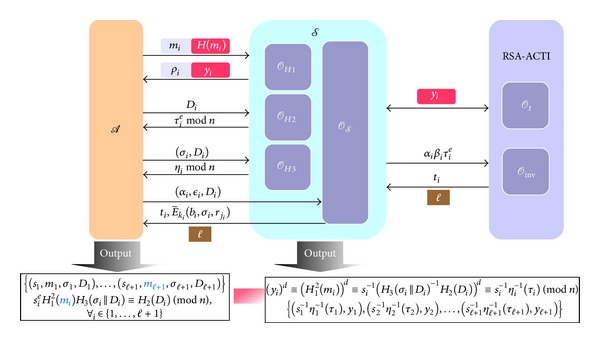
The proof model of FG-1.

**Figure 7:**
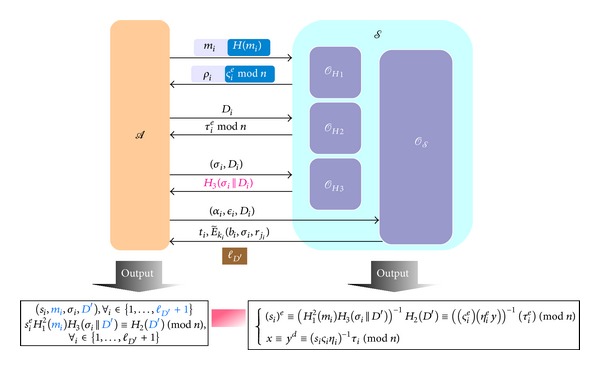
The proof model of FG-2.

Here we will start to do the simulation for the two games (i.e., FG-1 and FG-2) to prove *DAOECS* is secure against forgery attacks. The details of simulation are set below and illustrated in Figures 6 and 7, respectively.

*Simulation in FG-1*. In this proof model, *S* is allowed to query the oracles *O*_inv_ (i.e., (·)^*d*^) and *O*_*t*_ of RSA-ACTI problem defined in Definition 6 for helping *S* to produce e-cash(s) and the corresponding verifying key is (*e*, *n*).

- *H*_1_ Query of *O*_*H*_1__
- Initially, every blank record in *L*_*H*_1__ can be represented as (⊥, ⊥, ⊥). When *A* sends *m* for querying the hash value *H*_1_(*m*), *S* will check the list *L*_*H*_1__:
  - if *m* = *m*_*i*_ for some *i*, then *S* retrieves the corresponding *H*_1_(*m*_*i*_) and returns it to *A*;
  - else if *m* = *H*_1_(*m*_*i*_) and *H*_1_^2^(*m*_*i*_)≠⊥ for some *i*, then *S* retrieves the corresponding *H*_1_^2^(*m*_*i*_) and returns it to *A*;
  - else if *m* = *H*_1_(*m*_*i*_) and *H*_1_^2^(*m*_*i*_) =  ⊥ for some *i*, then *S* queries *O*_*t*_ to get an instance *y* and returns it to *A*, then fills the record (*m*_*i*_, *H*_1_(*m*_*i*_), ⊥) as (*m*_*i*_, *H*_1_(*m*_*i*_), *y*) in *L*_*H*_1__;
  - otherwise, *S* selects a random *ρ* ∈ *Z*_*n*_, records (*m*, *ρ*, ⊥) in *L*_*H*_1__, and returns *ρ* to *A*.
- *H*_2_ Query of *O*_*H*_2__
- When *A* asks for *H*_2_ query by sending *D* to *S*, *S* will look up the list *L*_*H*_2__:
  - if *D* = *D*_*i*_ for some *i*, the corresponding *τ* will be retrieved and *S* will send (*τ* ^*e*^mod⁡ *n*) back to *A*;
  - otherwise, *S* will select a random *τ* ∈ *Z*_*n*_, record (*D*, *τ*) in *L*_*H*_2__, and return (*τ* ^*e*^mod⁡ *n*) back to *A*.
- *H*_3_ Query of *O*_*H*_3__
- While *A* sends (*σ*, *D*) to *S* for *H*_3_(*σ*||*D*), *S* will look up the list *L*_*H*_3__:
  - if (*σ*, *D*) = (*σ*_*i*_, *D*_*i*_) for some *i*, the corresponding *η* will be retrieved and (*η* ^*e*^mod⁡ *n*) will be returned to *A*;
  - otherwise, *S* will select a random *η* ∈ *Z*_*n*_, record ((*σ*, *D*), *η*) in *L*_*H*_3__, and return (*η* ^*e*^mod⁡ *n*) back to *A*.
- E-Cash Producing Query of *O*_*S*_
- When *A* sends (*α*, *ϵ*, *D*) to *S*, *S* will do the following steps:
  1. decrypt *ϵ*, obtain (*k*, ID);
  2. randomly select *r*_*j*_ and prepare $\sigma ={\hat{E}}_{pk_{j}}(\text{ID}||r_{j})$;
  3. choose *η* ∈_*R*_ *Z*_*n*_, set *H*_3_(*σ*||*D*) = (*η* ^*e*^ mod *n*), and store ((*σ*, *D*), *η*) in *L*_*H*_3__;
  4. select *b* ∈_*R*_ *Z*_n_* and compute *β* = (*b* ^*e*^ *η* ^*e*^)^−1^ mod *n*;
  5. retrieve or assign *τ* such that *H*_2_(*D*) = (*τ* ^*e*^) as the *O*_*H*_2__ query described above;
  6. send (*αβτ* ^*e*^) to oracle *O*_inv_ to get *t* = (*αβτ* ^*e*^)^*d*^mod⁡ *n*;
  7. return $\left(t,{\tilde{E}}_{k}(b,\sigma ,r_{j})\right)$ back to *A*.

Eventually, assume that *A* can successfully output *l* + 1 e-cash tuples

(17) $$\begin{array}{c} \left\{\left(s_{1},m_{1},\sigma_{1},D_{1}\right)\cdots \left(s_{l+1},m_{l+1},\sigma_{l+1},D_{l+1}\right)\right\}, \end{array}$$

where *m*_*i*_ are all distinct, ∀*i*, 1 ≤ *i* ≤ *l* + 1, such that *s*_*i*_^*e*^ *H*_1_^2^(*m*)*H*_3_(*σ*_*i*_||*D*_*i*_) = *H*_2_(*D*_*i*_) (mod⁡ *n*) after *l* times to query *O*_*S*_ with nonnegligible probability *ϵ*_*A*_.

According to *L*_*H*_1__, *L*_*H*_2__, and *L*_*H*_3__, *S* can compute and retrieve RSA-inversion instances (∀*i*, 1 ≤ *i* ≤ *l* + 1)

(18) (yi)d≡(H12(mi))d≡si−1(H3(σi||Di)−1H2(Di))d≡si−1ηi−1(τi)(mod⁡ n).

Via *A* querying the signing oracle *O*_*S*_ for *l* times (i.e., query *O*_inv_ for *l* times by *S*), *S* can output *l* + 1 RSA-inversion instances

(19) {(s1−1η1−1(τ1),y1),(s2−1η2−1(τ2),y2),…,(sl+1−1ηl+1−1(τl+1),yl+1)}

and break the RSA-ACTI problem with nonnegligible probability at least *ϵ*_*A*_.

*Simulation in FG-2*. Initially, *S* is given an instance (*y*, *e*, *n*) of RSA inversion problem defined in Definition 7 and simulates the environment as follows.

- *H*_1_ Query of *O*_*H*_1__
- Initially, every blank record in *L*_*H*_1__ can be represented as (⊥, ⊥, ⊥). When *A* sends *m* for querying the hash value *H*_1_(*m*), *S* will check the list *L*_*H*_1__:
  - if *m* = *m*_*i*_ for some *i*, then *S* retrieves the corresponding *ρ*_*i*_ and returns it to *A*;
  - else if *m* = *H*_1_(*m*_*i*_) and *H*_1_^2^(*m*_*i*_)≠⊥ for some *i*, then *S* retrieves the corresponding *ς* and returns (*ς* ^*e*^mod⁡ *n*) to *A*;
  - else if *m* = *H*_1_(*m*_*i*_) and *H*_1_^2^(*m*_*i*_) =  ⊥ for some *i*, then *S* selects a random *ς* ∈ *Z*_*n*_, returns (*ς* ^*e*^mod⁡ *n*) to *A*, and then fills the record (*m*_*i*_, *H*_1_(*m*_*i*_), ⊥) as (*m*_*i*_, *H*_1_(*m*_*i*_), *ς*) in *L*_*H*_1__;
  - otherwise, *S* selects a random *ρ* ∈ *Z*_*n*_, records (*m*, *ρ*, ⊥) in *L*_*H*_1__, and returns *ρ* to *A*.
- *H*_2_ Query of *O*_*H*_2__
- When *A* asks for *H*_2_ query by sending *D* to *S*, *S* will look up the list *L*_*H*_2__:
  - if *D* = *D*_*i*_ for some *i*, the corresponding *τ* will be retrieved and *S* will send (*τ* ^*e*^mod⁡ *n*) back to *A*;
  - otherwise, *S* will select a random *τ* ∈ *Z*_*n*_, record (*D*, *τ*) in *L*_*H*_2__, and return (*τ* ^*e*^mod⁡ *n*) back to *A*.
- *H*_3_ Query of *O*_*H*_3__
- While *A* sends (*σ*, *D*) to *S* for *H*_3_(*σ*||*D*), *S* will look up the list *L*_*H*_3__:
  - if (*σ*, *D*) = (*σ*_*i*_, *D*_*i*_) for some *i*, the corresponding *H*_3_(*σ*_*i*_||*D*_*i*_) will be retrieved and returned to *A*;
  - otherwise, *S* will select a random *η* ∈ *Z*_*n*_, set *H*_3_(*σ*||*D*) = (*η* ^*e*^ *y* mod *n*), record ((*σ*, *D*), *η*, *H*_3_(*σ*||*D*)) in *L*_*H*_3__, and return *H*_3_(*σ*||*D*) back to *A*.
- E-Cash Producing Query of *O*_*S*_
- Let *l*_*D*_*i*__ be a counter to record the number of queries on each expiration date *D*_*i*_, which is initialized by 0. When *A* sends (*α*, *ϵ*, *D*) to *S*, *S* will do the following steps:
  1. decrypt *ϵ*, obtain (*k*, ID);
  2. randomly select *r*_*j*_ and prepare $\sigma ={\hat{E}}_{pk_{j}}(\text{ID}||r_{j})$;
  3. choose *η* ∈_*R*_ *Z*_*n*_, set *H*_3_(*σ*||*D*) = (*αη* ^*e*^mod⁡ *n*), and store ((*σ*, *D*), ⊥, (*αη* ^*e*^mod⁡ *n*)) and (*σ*, *D*) in *L*_*H*_3__ and *L*_*x*_, respectively;
  4. select *b* ∈_*R*_ *Z*_n_* and compute *β* = (*b* ^*e*^ *αη* ^*e*^)^−1^mod⁡ *n*;
  5. retrieve or assign *τ* such that *H*_2_(*D*) = (*τ* ^*e*^) as the *O*_*H*_2__ query described above;
  6. compute *t* ≡ (*αβτ* ^*e*^)^*d*^ ≡ ((*bη*)^−1^ *τ*) (mod⁡ *n*);
  7. set *l*_*D*_ = *l*_*D*_ + 1 and return $\left(t,{\tilde{E}}_{k}(b,\sigma ,r_{j})\right)$ back to *A*.

Eventually, assume that *A* can successfully output *l*_*D*′_ + 1 e-cash tuples for some expiration date *D*′

(20) $$\begin{array}{c} \left\{\left(s_{1},m_{1},\sigma_{1},D^{\prime }\right)\cdots \left(s_{l_{D^{\prime }}+1},m_{l_{D^{\prime }}+1},\sigma_{l_{D^{\prime }}+1},D^{\prime }\right)\right\} \end{array}$$

such that *s*_*i*_^*e*^ *H*_1_^2^(*m*_*i*_)*H*_3_(*σ*_*i*_||*D*′) = *H*_2_(*D*′) (mod⁡ *n*), ∀*i*, 1 ≤ *i* ≤ *l*_*D*′_ + 1, after *l*_*D*′_ times to query *O*_*S*_ on *D*′, with nonnegligible probability *ϵ*_*A*_.

Assume some (*σ*_*i*_, *D*′), 1 ≤ *i* ≤ *l*_*D*′_ + 1, is not recorded in *L*_*x*_; then by the *L*_*H*_1__, *L*_*H*_2__, and *L*_*H*_3__, *S* can compute and retrieve

(21) (si)e≡(H12(mi)H3(σi||D′))−1H2(D′)≡((ςie)(ηiey))−1(τie)(mod⁡ n),x≡yd≡(siςiηi)−1τi(mod⁡ n)

and solve the RSA inversion problem with nonnegligible probability at least *ϵ*_*A*_.

### 4.3. E-Cash Conditional-Traceability

**Figure 8:**
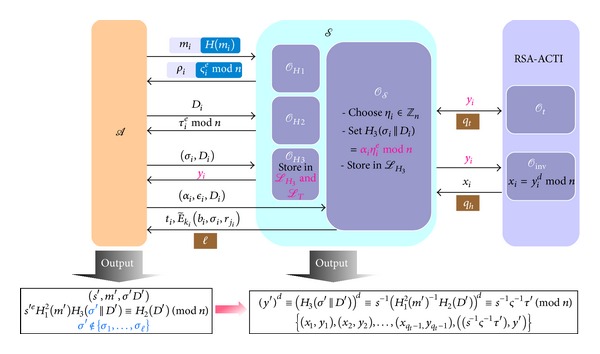
The proof model of TG.

In this section, we will prove that the ID information embedded in e-cash(s) cannot be replaced or moved out by any user against being traced after some misbehavior or criminals. The details of our proof model are illustrated in Figure 8.

**Definition 10 (Tampering Game (TG))**

Let *l*_*k*_ ∈ *N* be a security parameter and *A* be an adversary in *DAOECS*. *O*_*S*_ is an oracle which plays the role of bank in *DAOECS* to record parameters from the queries of *A* and issue e-cash(s) (i.e., (*s*, *m*, *σ*, *D*), where *m* = (*w*_1_, *y*_1_, *w*_2_, *y*_2_, *r*_3_, *D*)) accordingly. *A* is allowed to query *O*_*S*_ for *l* times; consider Algorithm 4.

*A* wins the game if the probability Pr[Exp_*A*_^TG^(*k*) = 1] of *A* is nonnegligible.

**Definition 11 (E-Cash Traceability)**

If there exists no probabilistic polynomial-time adversary who can win the tracing game TG, then *DAOECS* satisfies the E-Cash Traceability.

**Definition 12 (Alternative Formulation of RSA Known-Target Inversion Problem (RSA-AKTI))**

Let *k* ∈ *N* be a security parameter and *A* be an adversary who is allowed to access the RSA-inversion oracle *O*_inv_ and the target oracle *O*_*t*_. *A* is allowed to query *O*_*t*_ and *O*_inv_ for *q*_*t*_ and *q*_*h*_ times (*q*_*h*_ < *q*_*t*_), respectively. Consider Algorithm 5.

We say *A* breaks the RSA-AKTI problem if the probability Pr[Exp_*A*_^RSA-AKTI^(*k*) = 1] of *A* is nonnegligible.

**Theorem 13**

For a polynomial-time adversary *A* who can win the tracing game TG with nonnegligible probability, there exists another adversary *S* who can break the RSA-AKTI problem with nonnegligible probability.

**Proof**

*S* simulates the environment of *DAOECS* by controlling three hash oracles, *O*_*H*_1__, *O*_*H*_2__, *O*_*H*_3__, to respond hash queries and an e-cash producing oracle *O*_*S*_ of *DAOECS* to respond e-cash producing queries from *A*, respectively, in the random oracle model. Eventually, *S* will take advantage of *A*'s capability to solve RSA-AKTI problem. Then, for consistency, *S* maintains three lists *L*_*H*_1__, *L*_*H*_2__, and *L*_*H*_3__ to record every response of *O*_*H*_1__, *O*_*H*_2__, and *O*_*H*_3__, respectively.

Besides, in the proof model, *S* is allowed to query the oracles *O*_inv_ (i.e., (·)^*d*^) and *O*_*t*_ of the RSA-AKTI problem defined in Definition 12 for helping *S* produce valid e-cash(s) and the corresponding verifying key is (*e*, *n*).

Here we will do the simulation for game TG to prove that *DAOECS* satisfies the e-cash traceability. Details are described as follows.

- *H*_1_ Query of *O*_*H*_1__
- Initially, every blank record in *L*_*H*_1__ can be represented as (⊥, ⊥, ⊥). When *A* sends *m* for querying the hash value *H*_1_(*m*), *S* will check the list *L*_*H*_1__:
  - if *m* = *m*_*i*_ for some *i*, then *S* retrieves the corresponding *H*_1_(*m*_*i*_) and return it to *A*;
  - else if *m* = *H*_1_(*m*_*i*_) and *H*_1_^2^(*m*_*i*_)≠⊥ for some *i*, then *S* retrieves the corresponding *ς*_*i*_ and returns (*ς*_*i*_^*e*^mod⁡ *n*) to *A*;
  - else if *m* = *H*_1_(*m*_*i*_) and *H*_1_^2^(*m*_*i*_) =  ⊥ for some *i*, then *S* chooses *ς* ∈_*R*_ *Z*_*n*_, sets *H*_1_^2^(*m*_*i*_) = (*ς* ^*e*^mod⁡ *n*), and returns *H*_1_^2^(*m*_*i*_) to *A* then fills the original record (*m*_*i*_, *H*_1_(*m*_*i*_), ⊥) as (*m*_*i*_, *H*_1_(*m*_*i*_), *ς*) in *L*_*H*_1__;
  - otherwise, *S* selects a random *ρ* ∈ *Z*_*n*_, sets *H*_1_(*m*_*i*_) = *ρ*, records (*m*, *H*_1_(*m*_*i*_), ⊥) in *L*_*H*_1__, and returns *ρ* to *A*.
- *H*_2_ Query of *O*_*H*_2__
- When *A* asks for *H*_2_ query by sending *D* to *S*, *S* will look up the list *L*_*H*_2__:
  - if *D* = *D*_*i*_ for some *i*, the corresponding *τ* will be retrieved and *S* will send (*τ* ^*e*^mod⁡ *n*) back to *A*;
  - otherwise, *S* will select a random *τ* ∈ *Z*_*n*_, record (*D*, *τ*) in *L*_*H*_2__, and return (*τ* ^*e*^mod⁡ *n*) back to *A*.
- *H*_3_ Query of *O*_*H*_3__
- While *A* sends (*σ*, *D*) to *S* for *H*_3_(*σ*), *S* will look up the list *L*_*H*_3__:
  - if (*σ*, *D*) = (*σ*_*i*_, *D*_*i*_) for some *i*, the corresponding *y*_*i*_ will be retrieved and returned to *A*;
  - otherwise, *S* will query *O*_*t*_ to get an instance *y*; record *y* and ((*σ*, *D*), *y*) in *L*_*T*_ and *L*_*H*_3__, respectively;
  - return *y* back to *A*.
- E-Cash Producing Query of *O*_*S*_
- While *A* sends (*α*, *ϵ*, *D*) to *S*, *S* will do the following steps:
  1. decrypt *ϵ*, obtain (*k*, ID);
  2. randomly select *r*_*j*_ and prepare $\sigma ={\hat{E}}_{pk_{j}}(\text{ID}||r_{j})$;
  3. choose *η* ∈_*R*_ *Z*_*n*_, set *H*_3_(*σ*||*D*) = (*αη* ^*e*^mod⁡ *n*), and store ((*σ*, *D*), *H*_3_(*σ*||*D*)) in *L*_*H*_3__;
  4. select *b* ∈_*R*_ *Z*_n_* and compute *β* = (*b* ^*e*^ *αη* ^*e*^)^−1^mod⁡ *n*;
  5. retrieve or assign *τ* such that *H*_2_(*D*) = (*τ* ^*e*^) as the *O*_*H*_2__ query described above;
  6. compute *t* ≡ (*αβτ* ^*e*^)^*d*^ ≡ ((*bη*)^−1^ *τ*) (mod⁡ *n*);
  7. return $\left(t,{\tilde{E}}_{k}(b,\sigma ,r_{j})\right)$ back to *A*.

Assume that *A* can successfully output an e-cash tuples (*s*′, *m*′, *σ*′, *D*′), where *σ*′ never appeals as a part for some *O*_*S*_ query such that *s*′^*e*^ *H*_1_^2^(*m*′)*H*_3_(*σ*′||*D*′) ≡ *H*_2_(*D*′) (mod⁡ *n*); then by *L*_*H*_1__, *L*_*H*_2__, and *L*_*H*_3__, *S* can derive

(22) (y′)d≡(H3(σ′||D′))d≡s′−1(H12(m′)−1H2(D′))d≡s′−1ς′−1τ′(mod⁡ n).

Let |*L*_*T*_| = *q*_*t*_ and *L*_*T*_ = {*y*_1_,…, *y*_*q*_*t*__}. *S* sends *y*_*i*_ ∈ (*L*_*T*_ − {*y*′}), 1 ≤ *i* ≤ (*q*_*t*_ − 1), to *O*_inv_ and obtains *q*_*t*_ − 1  *x*_*i*_ such that *x*_*i*_ = *y*_*i*_^*d*^mod⁡ *n*.

Eventually *S* can output *q*_*t*_ RSA-inversion instances

(23) $$\begin{array}{c} \left\{\left(x_{1},y_{1}\right),\left(x_{2},y_{2}\right),\ldots ,\left(x_{q_{t}-1},y_{q_{t}-1}\right),\left(\left({s^{\prime }}^{-1}{\varsigma^{\prime }}^{-1}\tau^{\prime }\right),y^{\prime }\right)\right\} \end{array}$$

after querying *O*_inv_ for *q*_*h*_ times, where *q*_*h*_ = *q*_*t*_ − 1 < *q*_*t*_ and thus, it breaks the RSA-AKTI problem with nonnegligible probability at least *ϵ*_*A*_.

### 4.4. E-Cash No-Swindling

In typical online e-cash transactions, when an e-cash has been spent in previous transactions, another spending will be detected immediately owing to the double-spending check procedure. However, in an offline e-cash model, the merchant may accept a transaction involving a double-spent e-cash first and then do the double-spending check later. In this case, the original owner of the e-cash may suffer from loss. Therefore, a secure offline e-cash scheme should guarantee the following two events.

- No one, except the real owner, can spend a fresh and valid offline e-cash successfully.
- No one can double spend an e-cash successfully.

Roughly, it can be referred to as* e-cash no-swindling* property. In this section, we will define the no-swindling property and formally prove that our scheme is secure against swindling attacks.

**Definition 14 (Swindling Game in *DAOECS*)**

Let *l*_*k*_ ∈ *N* be a security parameter and *A* be an adversary in *DAOECS*. *O*_*B*_ is an oracle issuing generic e-cash(s) (i.e., (*s*, *y*_1_, *w*_1_, *x*_2_, *r*_2_, *r*_3_, *σ*, *D*)) of *DAOECS* to *A*. *O*_*off*⁡_ is an oracle to show the expanding form (*s*, *y*_1_, *w*_1_, *x*_2_, *r*_2_, *r*_3_, *σ*, *D*, *r*_*s*_, *s*′) for the payment according to the input (*s*, *m*, *σ*, *D*). Consider the two experiments SWG-1 and SWG-2 shown in Algorithms 6 and 7, respectively.

*A* wins the game if the probability Pr[Exp_*A*_^SWG-1^(*l*_*k*_) = 1] or Pr[Exp_*A*_^SWG-2^(*l*_*k*_) = 1] of *A* is nonnegligible.

**Definition 15 (E-Cash No-Swindling)**

If there exists no probabilistic polynomial-time adversary who can win the swindling game defined in Definition 14, then *DAOECS* satisfies e-cash no-swindling.

**Theorem 16**

For a polynomial-time adversary *A* who can win the swindling game SWG with nonnegligible probability, there exists another adversary *S* who can solve the discrete logarithm problem with nonnegligible probability.

**Proof**

Consider the swindling game defined in Definition 14. *S* simulates the environment by controlling the hash oracles, *O*_*H*_4__, to respond hash queries on *H*_4_ of *DAOECS* in the random oracle model. Eventually, *S* will take advantage of *A*'s capability to solve the discrete logarithm problem. Then, for consistency, *S* maintains a list *L*_*H*_4__ to record every response of *O*_*H*_4__. *S* is given all parameters (*pk*_*j*_, *sk*_*j*_, *g*_1_, *g*_2_, *e*_*b*_, *d*_*b*_, *p*_*b*_, *q*_*b*_, *n*_*b*_, *p*, *q*, *H*_1_, *H*_2_, *H*_3_, *H*_4_, *H*_5_) of *DAOECS* and an instance y* of discrete logarithm problem (i.e., y* = *g* ^x*^mod⁡ *p*). Here we will describe the simulations for the two experiments Exp_*A*_^SWG-1^ and Exp_*A*_^SWG-2^, individually.

**Figure 9:**
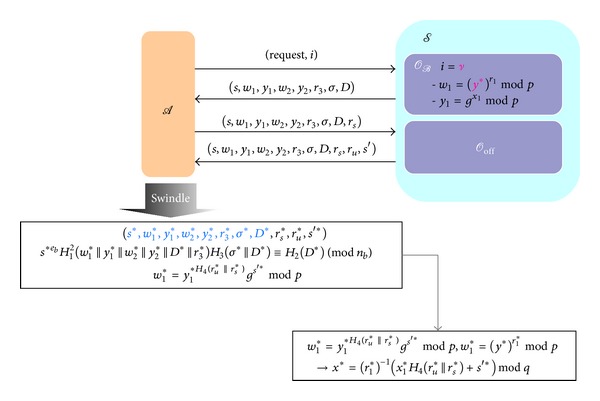
The proof model of SWG-1.

The simulation for Exp_*A*_^SWG-1^ is illustrated in Figure 9 and each oracle is constructed as follows.

- Oracle *O*_*B*_
- Initially, *S* guesses that the generic e-cash produced from *ν*th query will be the attack target. When *A* sends *i*th query to *O*_*B*_ for an e-cash, *O*_*B*_ will do the following:
  - select *r*_1_, *x*_1_, *r*_3_ ∈_*R*_ *Z*_*q*_ and *y*_2_, *w*_2_ ∈_*R*_ *Z*_*p*_;
  - if *i* = *ν*,
    - compute (*w*_1_ = (y*)^*r*_1_^mod⁡ *p*) and (*y*_1_ = *g* ^*x*_1_^mod⁡ *p*);
  - if *i* ≠ *ν*,
    - compute (*w*_1_ = *g* ^*r*_1_^mod⁡ *p*) and (*y*_1_ = *g* ^*x*_1_^mod⁡ *p*);
  - prepare *s* = ((*H*_1_^2^(*m*)*H*_3_(*σ*||*D*))^−1^ *H*_2_(*D*))^*d*_*b*_^mod⁡ *n*_*b*_, where *m* = (*w*_1_, *y*_1_, *w*_2_, *y*_2_, *r*_3_, *D*);
  - record (*i*, (*s*, *m*, *σ*, *D*), (*r*_1_, *x*_1_))) in list *L*_*B*_ and return (*s*, *m*, *σ*, *D*) to *A*.
- Oracle *O*_*off*⁡_
- When *A* sends a valid e-cash tuple (*s*, *w*_1_, *y*_1_, *w*_2_, *y*_2_, *r*_3_, *σ*, *D*, *r*_*s*_) to *O*_*off*⁡_, it will look up the list *L*_*B*_:
  - if (*s*, *w*_1_, *y*_1_, *w*_2_, *y*_2_, *r*_3_, *σ*, *D*) exists with prefix index *ν*, then abort;
  - otherwise, *O*_*off*⁡_ will retrieve the corresponding (*r*_1_, *x*_1_); choose a random *r*_*u*_, compute *u* = *H*_4_(*r*_*u*_||*r*_*s*_) and (*s*′ = *r*_1_ − *ux*_1_mod⁡ *q*), and send (*s*, *w*_1_, *y*_1_, *w*_2_, *y*_2_, *r*_3_, *σ*, *D*, *r*_*u*_, *r*_*s*_, *s*′) back to *A*.

Assume that *A* can successfully output a valid offline e-cash expansion tuple (s*, *w*_1_*, *y*_1_*, *w*_2_*, *y*_2_*, *r*_3_*, σ*, D*, *r*_u_*, *r*_s_*, *s* ^′∗^), where (s*, *w*_1_*, *y*_1_*, *w*_2_*, *y*_2_*, *r*_3_*, σ*, D*) is prefixed with *ν* and postfixed with (*r*_1_*, *x*_1_*) in *L*_*B*_. Then, since *w*_1_* = *y*_1_^∗*H*_4_(*r*_u_*||*r*_s_*)^ *g* ^*s*^′∗^^mod⁡ *p* and *w*_1_* = (y*)^*r*_1_*^, *S* can derive

(24) $$\begin{array}{c} x^{*}={\left(r_{1}^{*}\right)}^{-1}\left(x_{1}^{*}H_{4}\left(r_{u}^{*}||r_{s}^{*}\right)+s^{\prime *}\right)\mathrm{mod}q \end{array}$$

and solve the discrete logarithm problem with nonnegligible probability at least (1/*q*_*O*_*B*__)*ϵ*_*A*_, where *q*_*O*_*B*__ is the total number of *O*_*B*_ query.

**Figure 10:**
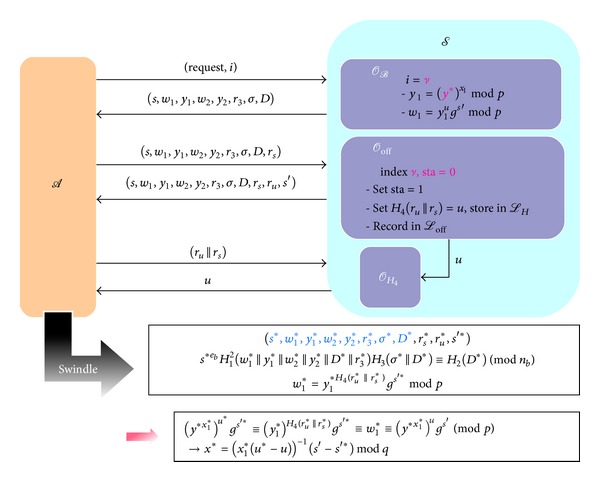
The proof model of SWG-2.

The simulation for Exp_*A*_^SWG-2^ is illustrated in Figure 10 and each oracle is constructed as follows.

- Oracle *O*_*B*_
- Initially, *S* guesses that the generic e-cash produced from *ν*th query will be the attack target. When *A* sends *i*th query to *O*_*B*_ for an e-cash, *O*_*B*_ will do the followings.
  - if *i* = *ν*:
    - select *s*′, *u*, *x*_1_, *r*_3_ ∈_*R*_ *Z*_*q*_ and *y*_2_, *w*_2_ ∈_*R*_ *Z*_*p*_;
    - compute (*y*_1_ = (y*)^*x*_1_^mod⁡ *p*) and (*w*_1_ = *y*_1_^*u*^ *g* ^*s*′^mod⁡ *p*);
    - prepare *s* = ((*H*_1_^2^(*m*)*H*_3_(*σ*||*D*))^−1^ *H*_2_(*D*))^*d*_*b*_^mod⁡ *n*_*b*_, where *m* = (*w*_1_, *y*_1_, *w*_2_, *y*_2_, *r*_3_, *D*);
    - record (*i*, (*s*, *m*, *σ*, *D*), (*u*, *s*′))) in list *L*_*B*_;
  - if *i* ≠ *ν*:
    - select *r*_1_, *x*_1_, *r*_3_ ∈_*R*_ *Z*_*q*_ and *y*_2_, *w*_2_ ∈_*R*_ *Z*_*p*_;
    - compute (*w*_1_ = *g* ^*r*_1_^mod⁡ *p*) and (*y*_1_ = *g* ^*x*_1_^mod⁡ *p*);
    - prepare *s* = ((*H*_1_^2^(*m*)*H*_3_(*σ*||*D*))^−1^ *H*_2_(*D*))^*d*_*b*_^mod⁡ *n*_*b*_, where *m* = (*w*_1_, *y*_1_, *w*_2_, *y*_2_, *r*_3_, *D*);
    - record (*i*, (*s*, *m*, *σ*, *D*), (*r*_1_, *x*_1_))) in list *L*_*B*_;
  - return (*s*, *m*, *σ*, *D*) to *A*.
- Oracle *O*_*off*⁡_
- A status parameter sta is initialized by 0. When *A* sends a valid e-cash tuple (*s*, *w*_1_, *y*_1_, *w*_2_, *y*_2_, *r*_3_, *σ*, *D*, *r*_*s*_) to *O*_*off*⁡_, it will look up the list *L*_*B*_:
  - if (*s*, *w*_1_, *y*_1_, *w*_2_, *y*_2_, *r*_3_, *σ*, *D*) exists with prefix index *ν* and sta = 0, *O*_*off*⁡_ will perform the following procedures:
    - set sta = 1
    - retrieve the corresponding (*u*, *s*′) from *L*_*B*_ and choose a random *r*_*u*_;
    - set *H*_4_(*r*_*u*_||*r*_*s*_) = *u* and record ((*r*_*u*_||*r*_*s*_), *u*) in *L*_*H*_;
    - record (*s*, *w*_1_, *y*_1_, *w*_2_, *y*_2_, *r*_3_, *σ*, *D*, *r*_*u*_, *r*_*s*_, *s*′) in list *L*_*off*⁡_;
    - send (*s*, *w*_1_, *y*_1_, *w*_2_, *y*_2_, *r*_3_, *σ*, *D*, *r*_*u*_, *r*_*s*_, *s*′) back to *A*;
  - if (*s*, *w*_1_, *y*_1_, *w*_2_, *y*_2_, *r*_3_, *σ*, *D*) exists with prefix index ≠*ν*, *O*_*off*⁡_ will retrieve the corresponding (*r*_1_, *x*_1_), choose random *r*_*u*_ and *u*, set *H*_4_(*r*_*u*_||*r*_*s*_) = *u*, record ((*r*_*u*_||*r*_*s*_), *u*) in *L*_*H*_, compute (*s*′ = *r*_1_ − *ux*_1_mod⁡ *q*), and send (*s*, *w*_1_, *y*_1_, *w*_2_, *y*_2_, *r*_3_, *σ*, *D*, *r*_*u*_, *r*_*s*_, *s*′) back to *A*.
  - Otherwise, abort.
- Oracle *O*_*H*_4__
- While *A* sends (*r*_*u*_||*r*_*s*_) to query for *H*_4_(*r*_*u*_||*r*_*s*_), *O*_*H*_4__ will check the list *L*_*H*_:
  - if (*r*_*u*_||*r*_*s*_) exists as the prefix of some record, *O*_*H*_4__ will retrieve the corresponding *u* and return it to *A*;
  - otherwise, *O*_*H*_4__ will choose a random *u*, record ((*r*_*u*_||*r*_*s*_), *u*) in *L*_*H*_, and return *u* to *A*.

Assume that *A* can successfully output a valid offline e-cash expansion tuple (s*, *w*_1_*, *y*_1_*, *w*_2_*, *y*_2_*, *r*_3_*, σ*, D*, *r*_u_*, *r*_s_*, *s* ^′∗^), where (s*, *w*_1_*, *y*_1_*, *w*_2_*, *y*_2_*, *r*_3_*, σ*, D*) is prefixed with *ν* and postfixed with (*u*, *s*′) in *L*_*B*_ and *H*_4_(*r*_u_*||*r*_s_*) ≠ *u*.

Then, via *L*_*H*_, since

(25) (y∗x1∗)u∗gs′∗≡(y1∗)H4(ru∗||rs∗)gs′∗≡w1∗≡(y∗x1∗)ugs′(mod⁡p),

*S* can derive

(26) $$\begin{array}{c} x^{*}={\left(x_{1}^{*}\left(u^{*}-u\right)\right)}^{-1}\left(s^{\prime }-s^{\prime *}\right)\mathrm{mod}q \end{array}$$

and solve the discrete logarithm problem with nonnegligible probability at least (1/*q*_*O*_*B*__)*ϵ*_*A*_, where *q*_*O*_*B*__ is the total number of *O*_*B*_ query.

Summarize the proof models for the two experiments shown above, if there exists a polynomial-time adversary who can win the swindling game with nonnegligible probability, then there exists another one who can solve the discrete logarithm problem with nonnegligible probability. It implies that there exists no p.p.t. adversary who can win the swindling game, and our proposed offline e-cash scheme *DAOECS* satisfies no-swindling property.

## 5. E-Cash Advanced Features and Performance Comparisons

In this section, we compare the e-cash features and performance of our proposed scheme with other schemes given in [9, 13–15, 21, 22, 27, 38–40]. We analyze the features and performance of the aforementioned schemes and form a table (Table 1) for the summary.

### 5.1. Features Comparisons

All the schemes mentioned above fulfill the basic security requirements stated in Section 1, which are anonymity, unlinkability, unforgeability, and no double-spending. Besides these features, there can be other advanced features on an e-cash system discussed in the literatures. We focus on three other advanced features, which are traceability, date attachability, and no-swindling, and we compare the proposed scheme with the aforementioned schemes.

We also propose an e-cash renewal protocol for users to exchange a new valid e-cash with their unused but expired e-cash(s); therefore, users do not have to deposit the e-cash before it expires and withdraw a new e-cash again. Our proposed e-cash renewal protocol reduces the computation cost by 49.5% as compared to withdrawal and deposit protocols, which is almost half of the effort of getting a new e-cash, at the user side. It does a great help to the users since their devices usually have a weaker computation capability, such as smart phones.

### 5.2. Performance Comparisons

According to [41], we can summarize and induce the computation cost of all operations as follows. The computation cost of a modular exponentiation computation is about 240 times of the computation cost of a modular multiplication computation, while the computation cost of a modular inversion almost equals to that of a modular exponentiation. Also, the computation cost of a hash operation almost equals to that of a modular multiplication.

With the above assumptions, the total computation cost of users during withdrawal and payment phases of our proposed scheme can be induced as 1452 times of a modular multiplication computation, while other works [9, 13–15, 21, 22, 27, 38–40] need 3375, 1448, 5534, 966, 1450, 480, 4337, 7468, 5291, and 1449 times of a modular multiplication computation to finish withdrawal and payment phases at the user ends.

According to [15], we assume the RSA parameters *n*, *p*, *q* are 1024, 512, and 512 bits, respectively. We adopt AES and SHA-1 as the symmetric cryotsystem and one-way hash function used in all protocols, respectively; therefore, the signed message and hash massage are in 128 and 160 bits, respectively. We assume the expiration date is in 32 bits.

With the above assumptions, we compute the communication cost of each offline transaction, withdrawal, and payment, at the user side. Our scheme needs 2048 bits for withdrawing an e-cash and 6688 bits for spending an e-cash, which is 1092 bytes for each transaction.

The details of the comparisons are summarized in Table 1.

## 6. Conclusion

In this paper, we have presented earlier a provably secure offline electronic cash scheme with an expiration date and a deposit date attached to it. Besides, we have also designed an e-cash renewal protocol, where users can exchange their unused and expired e-cash(s) for new ones more efficiently. Compared with other similar works, our scheme is efficient from the aspect of considering computation cost of the user side and satisfying all security properties, simultaneously. Except for anonymity, unlinkability, unforgeability, and no double-spending, we also formally prove that our scheme achieves conditional-traceability and no-swindling. Not only does our scheme help the bank to manage their huge databases against unlimited growth, but also it strengthens the preservation of users' privacy and rights as well.

## Figures and Tables

**Algorithm 1:**
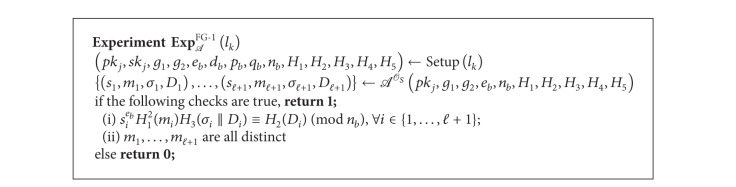
Experiment FG-1.

**Algorithm 2:**
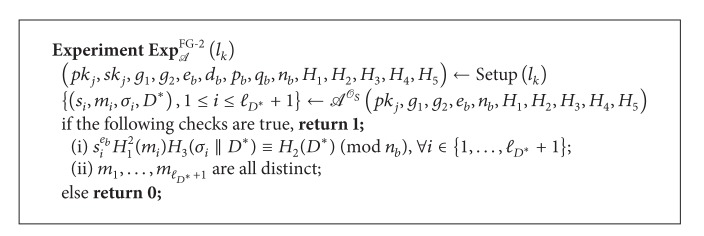
Experiment FG-2.

**Algorithm 3:**
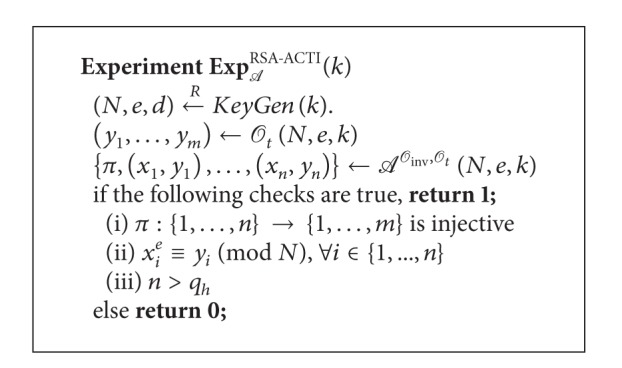


**Algorithm 4:**
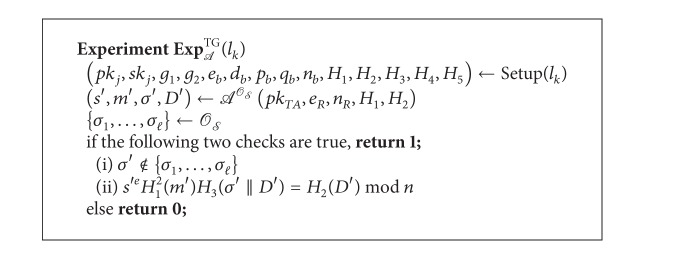


**Algorithm 5:**
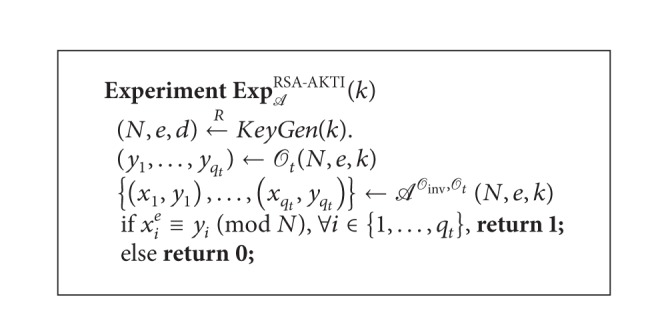


**Algorithm 6:**
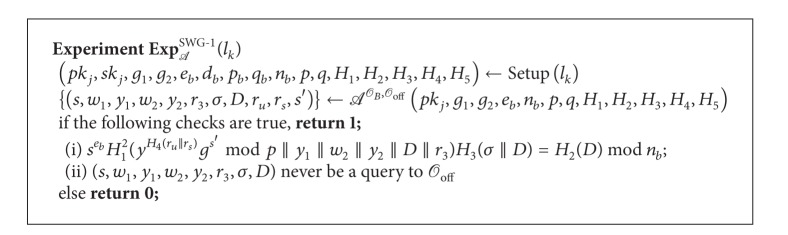
Experiment SWG-1.

**Algorithm 7:**
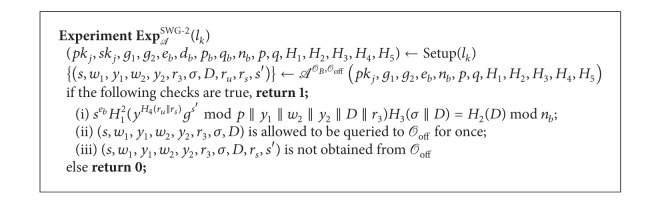
Experiment SWG-2.

**Table 1:** Advanced features and performance comparisons.

	Ours	[38]	[14]	[15]	[9]	[21]	[22]	[39]	[40]	[13]	[27]
Advanced features											
On/off-line	Off	Off	Off	Off	On	Off	Off	Off	Off	On	Off
Conditional-traceability	Yes	Yes	No	Yes	No	Yes	Yes	Yes	Yes	Yes	No
Date attachability	Yes	No	No	No	Yes	Yes	No	No	No	No	Yes
No-swindling	Yes	No	No	No	—	No	Yes	No	No	—	No
Renewal protocol	Yes	—	Yes	—	No	Yes	Yes	—	—	—	Yes
Formal proof	Yes	Yes	No	Yes	No	No	Yes	Yes	Yes	Yes	No

Performance											
Transaction cost^*⋆*^	5*E* + 7*M* +7*H* + 1inv +1*A* ≈1454*M*	14*E* + 14*M* +1*H* + 5*A* ≈3375*M*	6*E* + 8*M* ≈1448*M*	23*E* + 14*M* +1*A* ≈5534*M*	2*E* + 2*M* +2*H* ≈966*M*	5*E* + 9*M* +1*H* + 1inv +2*A* ≈1450*M*	2*E* ≈ 480*M*	18*E* + 15*M* +2*H* + 8*A* ≈4337*M*	31*E* + 22*M* +6*H* + 10*A* ≈7468*M*	22*E* + 11*M* +4*A* ≈5291*M*	6*E* + 8*M* +1*H* ≈1449*M*

Communication cost^*⋄*^	1092	576	1288	939	769	644	300	828	968	1536	728

According to [41], *H* ≈ *M*, *E* ≈ inv ≈ 240*M*.

*E*: a modular exponentiation; *M*: a modular multiplication; *H*: a hash operation; zkp: a zero-knowledge proof.

*A*: a modular addition; inv: a modular inversion.

^*⋆*^The computation cost of withdrawal and payment protocols at user side.

^*⋄*^The communication cost of each transaction at user side in bytes.
